# An Update on the Evolution of Glucosyltransferase (*Gtf*) Genes in *Streptococcus*

**DOI:** 10.3389/fmicb.2018.02979

**Published:** 2018-12-04

**Authors:** Rong-Rong Xu, Wei-Dong Yang, Ke-Xin Niu, Bin Wang, Wen-Mei Wang

**Affiliations:** ^1^Nanjing Stomatological Hospital, Nanjing University Medical School, Nanjing, China; ^2^Laboratory of Plant Genetics and Molecular Evolution, School of Life Sciences, Nanjing University, Nanjing, China

**Keywords:** *Streptococcus*, glucosyltransferase gene (*Gtf*), evolution, duplication, selection

## Abstract

In many caries-promoting *Streptococcus* species, glucosyltransferases (Gtfs) are recognized as key enzymes contributing to the modification of biofilm structures, disruption of homeostasis of healthy microbiota community and induction of caries development. It is therefore of great interest to investigate how *Gtf* genes have evolved in *Streptococcus.* In this study, we conducted a comprehensive survey of *Gtf* genes among 872 *streptococci* genomes of 37 species and identified *Gtf* genes from 364 genomes of 18 species. To clarify the relationships of these *Gtf* genes, 45 representative sequences were used for phylogenic analysis, which revealed two clear clades. Clade I included 12 *Gtf* genes from nine caries-promoting species of the Mutans and Downei groups, which produce enzymes known to synthesize sticky, water-insoluble glucans (WIG) that are critical for modifying biofilm structures. Clade II primarily contained *Gtf* genes responsible for synthesizing water-soluble glucans (WSG) from all 18 species, and this clade further diverged into three subclades (IIA, IIB, and IIC). An analysis of 16 pairs of duplicated *Gtf* genes revealed high divergence levels at the C-terminal repeat regions, with ratios of the non-synonymous substitution rate (dN) to synonymous substitution rate (dS) ranging from 0.60 to 1.03, indicating an overall relaxed constraint in this region. However, among the clade I *Gtf* genes, some individual repeat units possessed strong functional constraints by the same criterion. Structural variations in the repeat regions were also observed, with detection of deletions or recent duplications of individual repeat units. Overall, by establishing an updated phylogeny and further elucidating their evolutionary patterns, this work enabled us to gain a greater understanding of the origination and divergence of *Gtf* genes in *Streptococcus*.

## Introduction

The bacterial genus *Streptococcus* comprises many species living in human or animal oral cavities, which are collectively called oral *streptococci* (Coykendall, 1989; Whiley and Beighton, 1998; Facklam, 2002; Kohler, 2007). According to recent phylogenomic studies (Shao et al., 2013; Gao et al., 2014; Richards et al., 2014), the genus *Streptococcus* includes at many as eight monophyletic groups, and the oral *streptococci* are mainly distributed in six of these groups: Mitis, Sanguinis, Anginosus, Mutans, Salivarius, and Downei, each of which is named with a representative species. Among these, the Mutans and Downei groups are known to contain many caries-promoting species, which are normally minor in oral microbiome under healthy condition but can grow to markedly higher proportions under disease condition (Loesche, 1986; Whiley and Beighton, 1998; Balakrishnan et al., 2000; Richards et al., 2014; Kilian et al., 2016; Sanz et al., 2017; Manji et al., 2018; Philip et al., 2018; Twetman, 2018).

As the representative species of the Mutans group, *Streptococcus mutans* is widely recognized as a caries-promoting species in human oral cavity (Cornejo et al., 2013; Lemos et al., 2013). The involvement of *S. mutans* in caries development includes three phases. First, the bacteria can efficiently adhere to the surfaces of teeth and promote the formation of an extracellular, multi-dimensional structure known as a dental biofilm. Under health condition, a wide diversity of oral microbes dwells in the dental biofilm and the number of caries-promoting bacteria like *S. mutans* is maintained at low level. Second, when frequently exposed to fermentable carbohydrates in a diet, a large quantity of acidic products would be generated and released into the extracellular matrix and bacteria like *S. mutans* would be favored by lower pH environment in the biofilm and the growth of other oral bacteria would be inhibited. Third, once a dominant status of acid-producing and acid-tolerating bacteria like *S. mutans* is established within the biofilm, the acidic products would accumulate and promote the demineralization of tooth enamel, and eventually form dental caries (Lemos et al., 2013; Mclean, 2014; Guo et al., 2015; Kilian et al., 2016; Manji et al., 2018). In addition to *S. mutans*, another caries-promoting species in human is *S. sobrinus* of the Downei group, which is more frequently isolated from teenagers and children (Okada et al., 2005, 2012). Many other caries-promoting species in the Mutans and Downei groups are instead associated with various dentate mammals. These includes *S. macacae* and *S. downei* recovered from dental plaques of monkeys (Beighton et al., 1984; Whiley et al., 1988), *S. troglodytae* from oral cavities of chimpanzees (Okamoto et al., 2013), *S. ratti* and *S. criceti* from caries lesions of rodents (Coykendall, 1989), *S. dentirousetti* from oral cavities of fruit bats (Takada and Hirasawa, 2008), *S. orisuis* from the mouths of pigs (Takada and Hirasawa, 2007), and *S. devriesei* and *S. orisasini* isolated from horse and donkey teeth, respectively (Collins et al., 2004; Takada et al., 2013).

In the Salivarius group, animal experiments have shown that some strains of *S. salivarius* are capable of promoting high levels of caries, while another species, *S. vestibularis*, seems to lack this ability (Drucker et al., 1984; Willcox et al., 1991). The oral *Streptococci* in the Mitis, Sanguinis, and Anginosus groups are usually not regarded as caries-promoting species; rather, many of these species cause severe human diseases if they gain entrance to sites that are usually sterile (Coykendall, 1989; Whiley and Beighton, 1998; Facklam, 2002; Kohler, 2007). For example, *S. pneumoniae*, which normally reside in the human upper respiratory tract, is a leading cause of life-threatening diseases, such as sepsis, meningitis, and pneumonia (O’Brien and Nohynek, 2003).

Studies of several caries-promoting *Streptococcus* species have identified glucosyltransferases (Gtfs, also known as dextransucrases, EC 2.4.1.5) as critical enzymes contributing to caries development (Mukasa et al., 1985; Yamashita et al., 1993; Colby et al., 1999; Bowen and Koo, 2011; Koo et al., 2013; Krzysciak et al., 2014). Belonging to the glycoside hydrolase family 70 (GH70), Gtf enzymes and their close homologs are found in several lactic acid bacteria genera, including *Streptococcus, Leuconostoc, Lactobacillus*, and *Weissella* (Leemhuis et al., 2013). Having a conserved, central catalytic GH70 domain (for sucrose binding and splitting) and a variable, C-terminal repeat region (for potential glucan binding), these enzymes are able to synthesize a variety of α-glucans, such as dextran, mutan, and alternan from sucrose (Monchois et al., 1999). Early studies of *S. mutans* strain GS-5 have identified three different Gtf enzymes, GtfB, GtfC, and GtfD (Shiroza et al., 1987; Ueda et al., 1988; Honda et al., 1990). Recent studies have shown that these enzymes play differential roles in modifying biofilm structures toward caries development (Bowen and Koo, 2011; Krzysciak et al., 2014). GtfB, which mainly synthesizes sticky, water-insoluble glucans (WIG) rich in α1, 3-linkages, can adsorb onto the surfaces of *S. mutans* as well as other oral bacteria and promote the aggregation of bacterial cells in biofilm. Instead, GtfC exhibits a high affinity to tooth enamel pellicles and greatly helps bacteria adhere onto tooth surfaces by synthesizing a mixture of WIG and water-soluble glucans (WSG, rich in α1, 6-linkages). GtfB and GtfC are also involved in concentrating protons around *S. mutans* and pre-conditioning of bacteria for the acidic environments in biofilms (Guo et al., 2015). Predominantly synthesizing soluble glucans, GtfD is known to serve as primers for GtfB to enhance the overall synthesis efficiency of glucans (Bowen and Koo, 2011; Krzysciak et al., 2014). Overall, the biofilm matrix rich in WIG is more porous (Dibdin and Shellis, 1988), and favors carbohydrate diffusion through the matrix, allowing even the deepest bacteria in the biofilm (near tooth surface) to ferment carbohydrate and release acids.

Like *S. mutans*, other caries-promoting *Streptococci* would also rely on certain WIG-synthesizing Gtfs to influence the biofilm structures, creating an environment favoring caries development. In species that are not regarded as caries-promoting, such as *S. gordonii, S. oralis*, and *S. sanguinis*, only one type of Gtf enzyme synthesizing soluble glucans could be found, with their genes designated *GtfG, GtfR*, and *GtfP*, respectively (Vickerman et al., 1997; Fujiwara et al., 2000; Vacca Smith et al., 2000; Xu et al., 2007). However, multiple types of Gtf enzymes synthesizing both WSG and WIG have been isolated from caries-promoting species. Specifically, two Downei group species, *S. downei* and *S. sobrinus*, have been reported to produce four different types of Gtf enzymes encoded by genes *GtfI, GtfS, GtfT*, and *GtfU* (Ferretti et al., 1987; Yamashita et al., 1989; Gilmore et al., 1990; Abo et al., 1991; Hanada et al., 1993, 2002; Colby et al., 1999). Two of these genes, *GtfS* and *GtfT*, were also found arranged in tandem on chromosomes in *S. criceti* and *S. dentirousetti* (Inoue et al., 2000; Shinozaki-Kuwahara et al., 2013). Moreover, four different *Gtf* genes, namely *GtfJ, GtfK, GtfL*, and *GtfM*, were identified in *S. salivarius* ATCC 25975 (Giffard et al., 1991, 1993; Simpson et al., 1995b). Examining these Gtf enzymes revealed that, similar to *S. mutans* GtfB and GtfC, *S. downei* GtfI, *S. sobrinus* GtfI, and *S. salivarius* GtfJ and GtfL all synthesize WIG, while other Gtfs, similar to *S. mutans* GtfD, mainly synthesize WSG (Russell et al., 1987, 1990; Abo et al., 1991; Hanada et al., 1991; Simpson et al., 1995a; Nanbu et al., 2000).

Wondering whether Gtf enzymes synthesizing insoluble glucans in different *Streptococcus* species would be closely related, Giffard et al. (1993) and Simpson et al. (1995b) reconstruct distance trees using 8 and 12 *Streptococcus Gtf* genes, respectively. Surprisingly, they found that although *S. salivarius GtfJ* and *GtfL* are responsible for synthesizing insoluble glucans, they are not actually closely related to *GtfB* and *GtfC* of *S. mutans* and *GtfIs* of Downei group species. Later, Hoshino et al. (2012) investigated *Streptococcus Gtf*s as well as their homologs in *Leuconostoc* and *Lactobacillus* and found that all *Streptococcus Gtfs* share a common ancestor. They also proposed that the ancestral *Streptococcus Gtf* gene was likely acquired from a lactic acid bacterium via horizontal gene transfer (HGT). As more *Gtf* genes from various *Streptococcus* species became available, Argimon et al. (2013) further reported a phylogeny covering 39 *Gtf* sequences from 16 *Streptococcus* species. The results revealed for the first time that *Streptococcus Gtfs* had diverged into two major clades, with one comprising only Gtfs synthesizing WIG and the other including mainly Gtfs synthesizing WSG. The *S. salivarius GtfJ* and *GtfL* originated within the WSG clade, and it is believed that their abilities to synthesize WIG were acquired secondarily, probably because of selective divergence after gene duplication (Argimon et al., 2013). The *Gtf* gene phylogenies reported by both Hoshino et al. (2012) and Argimon et al. (2013) are based on the conserved GH70 domain sequences. However, on one hand, the range of GH70 domain is often not clearly defined among these studies. On the other hand, recombination events leading to incorrect topology likely occur on conserved GH70 domain region. So far, it remains largely unexplored whether the C-terminal repeat regions of *Gtf* genes can be included for phylogenetic inferences.

Taken together, previous studies have shown a general picture of the evolutionary history of *Gtf* genes in *Streptococcus*, but many aspects are still vague. For example, it is often unclear how many different types of *Gtf* genes are present in a given *Streptococcus* species. In a recent genome comparison study (Conrads et al., 2014), up to seven different *Gtf* genes (some only represent partial genes) were reported in *S. sobrinus* DSM 20742 compared to three in *S. mutans* UA159 (Ajdić et al., 2002). Due to increased availability of genome sequences of *Streptococcus* species/strains in recent years, it is now feasible to define *Gtf* gene numbers accurately among different *Streptococcus* species. Moreover, although previous phylogenetic studies have identified two major clades of *Gtf* genes (one WIG clade and the other WSG clade), further subgrouping of *Gtf* genes within clades and their relationships among various *Streptococcus* groups have not been elucidated well. Additionally, the presence of multiple *Gtf* genes in certain *Streptococcus* species suggests that many gene duplication events have occurred; however, the divergence patterns of duplicated *Gtf* genes, especially at variable C-terminal repeat regions, have not been systematically investigated. To gain a better understanding of *Gtf* gene evolution in *Streptococcus*, we surveyed a total of 872 *Streptococci* genomes covering 37 species (both cariogenic and non-cariogenic) to detect *Gtf* genes. We then used 45 representative sequences from 18 species or 317 sequences from 157 strains to establish robust *Gtf* gene phylogenies, which revealed more elaborate evolutionary relationships of *Gtf* genes from various *streptococcus* groups or species. More importantly, after speciation events or duplication events, the divergence patterns of *Gtf* genes, especially at the C-terminal repeat regions, were thoroughly examined.

## Materials and Methods

### *Streptococcus* Species Surveyed in This Study

Three recent phylogenomic studies have established a rather stable *Streptococcus* phylogeny, in which *Streptococcus* species were assigned into as many as eight monophyletic groups (Shao et al., 2013; Gao et al., 2014; Richards et al., 2014). Among these, the Anginosus and the newly proposed Sanguinis groups are sisters to each other, and they together with the Mitis group form a monophyletic clade (the ASM clade). The remaining five groups form the second monophyletic clade (SDMBP clade), with the Salivarius group sister to the new Downei group and the Bovis group sister to the Pyogenic group. The relationships among the Salivarius-Downei groups, the Mutans group, and the Bovis-Pyogenic groups have not yet been resolved, although one study supported a closer relationship between the Mutans group and the Bovis-Pyogenic groups (Gao et al., 2014). In addition, the *S. suis* species lies at an independent position between the two clades.

**FIGURE 1:**
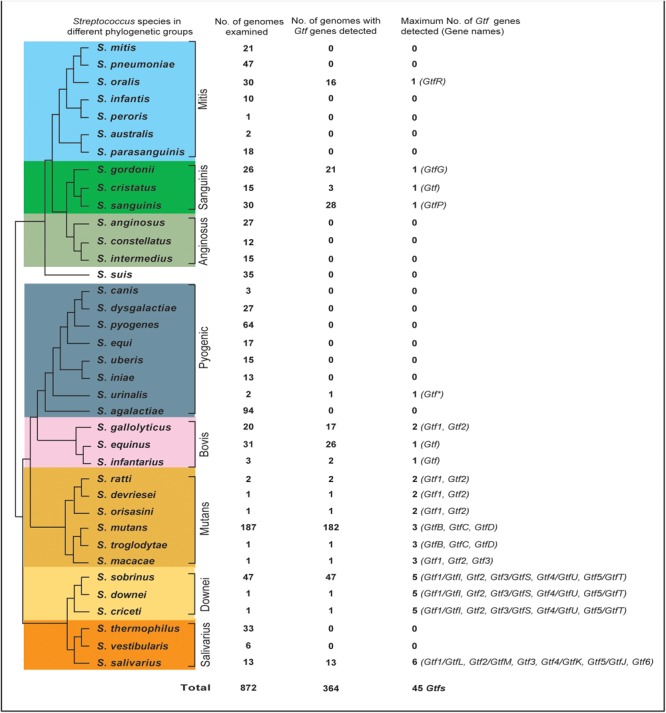
The distribution of Glucosyltransferase (*Gtf*) genes among 37 surveyed *Streptococcus* species. The phylogenetic relationships of streptococci were drawn based on three recent phylogenomic studies (Shao et al., 2013; Gao et al., 2014; Richards et al., 2014). A total of 872 genomes for the 37 species were examined, with 1 to 6 types of *Gtf* genes detected in 364 genomes of 18 species.

According to the phylogeny, a total of 37 *Streptococcus* species were selected for this study (Figure 1). These include seven species from the Mitis group (*S. mitis, S. pneumoniae, S. oralis, S. infantis, S. peroris, S. australis*, and *S. parasanguinis*), three species from the Sanguinis group (*S. gordonii, S. cristatus*, and *S. sanguinis*), three species from the Anginosus group (*S. anginosus, S. constellatus*, and *S. intermedius*), the independent species *S. suis*, eight species from the Pyogenic group (*S. canis, S. dysgalactiae, S. pyogenes, S. equi, S. uberis, S. iniae, S. urinalis*, and *S. agalactiae*), three species from the Bovis group (*S. gallolyticus, S. equinus*, and *S. infantarius*), six species from the Mutans group (*S. ratti, S. devriesei, S. orisasini, S. mutans, S. troglodytae*, and *S. macacae*), three species from the Downei group (*S. criceti, S. downei*, and *S. sobrinus*), and three species from the Salivarius group (*S. thermophilus, S. vestibularis*, and *S. salivarius*).

### Surveying *Gtf* Genes Among *Streptococci* Species

The genome sequence(s) available on the NCBI (National Center for Biotechnology Information) Microbial Genomes resource site^1^ on January 1, 2018 were surveyed for each of the 37 *Streptococcus* species. When there were many genome sequences available for a given species, those with better assembly levels were always examined first since these genomes have a higher chance to have a complete set of Gtf genes annotated. Overall, a total of 872 genomes were surveyed, and their detailed information is provided in Supplementary Table S1. For each genome, all of its protein sequences were downloaded and searched via BLASTP using the *S. mutans* UA159 Gtfs (NC_004350, SMU_910; SMU_1004; SMU_1005) as query sequences. The positive hits with *E*-values smaller than 1e-6 and query coverage higher than 50% were taken as potential Gtfs, which were further subjected to domain analyses on the NCBI Conserved Domain search site^2^. Only those possessing both the central GH70 domain and also the C-terminal glucan-binding domain (GBD) repeat regions were considered authentic Gtf enzymes for further analysis (Supplementary Table S1). In each species, the maximum number of *Gtf* genes detected from a representative strain was determined and these genes were named numerically (as *Gtf1, Gtf2, Gtf3*…). For genes detected in other strains of the same species, the detected genes were named accordingly by performing sequence comparisons and/or phylogenetic analyses (see next section). If a specific name was previously assigned to a *Gtf* gene in the literature, then the name was listed as well.

### Reconstructing the Phylogenies of *Streptococcus Gtf* Genes

Five outgroup sequences were selected to represent the anciently diverged lineages that were homologous to *Streptococcus Gtf* genes in this study: *Weissella confusa* strain VTT E-90392 dextransucrase gene (KJ173611, 4272bp), *Leuconostoc mesenteroides* dextransucrase gene *DsrD* (AY017384, 4584bp), *Lactobacillus parabuchneri* strain 33 glucansucrase gene (AY697432, 4686bp), *Lactobacillus sucicola* JCM 15457 *GtfG* gene (GAJ25773, 4752bp), and *Leuconostoc mesenteroides* subsp. *mesenteroides* strain BD3749 *Gtf* gene (KU306934, 4440bp).

To clarify the relationships of the identified *Streptococcus Gtf* genes, one representative strain for each species was selected first (Supplementary Table S1), and a data matrix consisting of 45 different *Gtf* genes from 18 *Streptococcus* species was compiled. Using the ClustalW and Muscle algorithms implemented in MEGA 5.0 (Tamura et al., 2011), these genes were aligned with the five outgroup genes using their protein sequences as a guide. By examining the obtained alignment in terms of homology, two major variable regions were identified. The first variable region (sites 139–966) is located between the N-terminal signal peptide region (SP) and a short conserved region (SCR) upstream of the central catalytic GH70 domain, and the second variable region (sites 4698–6897 bp) is at the C-terminal repeat region (for details, see next section and Supplementary Table S2). Since variable regions unlikely represent authentically homologous sites, these two regions were removed. The remaining alignment (∼3.9 kb) includes four rather conserved regions: the SP region, the SCR region, the entire GH70 domain, and the first two glucan-binding domain repeat units at the C-terminal repeat region (Supplementary Table S2). The 3.9 kb alignment was then used to reconstruct a Maximum-Likelihood (ML) tree in MEGA 5.0 by choosing the most appropriate nucleotide substitution model (GTR+I+ Γ), with 100 bootstrap replicates performed to test the robustness of internal branches (Tamura et al., 2011). Major clades or subclades were identified by examining the obtained phylogeny. Meanwhile, to explore the contribution of different regions to the phylogenetic reconstruction, two additional phylogenies were reconstructed by using either a 3.1 kb alignment covering the SP, SCR, and GH70 domain regions or a 2.6 kb alignment covering only the GH70 domain. Finally, to double-examine the reconstructed relationships and also to avoid any possible shortcomings incurred by using only one representative strain for a species, the sequence data from multiple strains was used to reconstruct the *Gtf* gene phylogenies for each major clade or subclade individually. In a total, 317 sequences from 157 strains of the 18 species were used.

### Analysis of the Divergence Patterns of *Streptococcus Gtf* Genes

For all 45 *Streptococcus* Gtf enzymes as well as the 5 outgroup proteins, the positions for various domains were carefully determined through the NCBI conserved domain searches and manual examination of the alignment. These domains included the N-terminal SP region (∼40 aa), a SCR region (∼100 aa) upstream of the GH70 domain, the conserved catalytic GH70 domain (∼800 aa), and the C-terminal repeat region (∼220–500 aa). Within the repeat regions, various numbers of Glucan-binding domain (GBD, TIGR04035, ∼65aa) repeat units were further determined. Since these GBD units all start with a conserved A repeat sequence (∼33 aa, Gilmore et al., 1990; Shah et al., 2004), the positions for all A repeating units were determined for each Gtf enzyme.

To explore the divergence patterns of *Gtf* genes after duplication events, sixteen pairs of *Gtf* genes were analyzed. For each pair, the genetic distances for the full-length gene (D*_*gene*_*), the conserved GH70 domain (D*_*GH70*_*), and the C-terminal repeat regions (D*_*repeat region*_*) were calculated in MEGA 5.0 (Tamura et al., 2011) by selecting the Tamura-Nei nucleotide substitution model and pairwise deletion. To also examine the selection pressures acting on the GH70 domain and the C-terminal repeat regions, the ratios of the non-synonymous substitution rate (Dn) to synonymous substitution rate (Ds) were calculated with the modified Nei-Gojobori method (Proportion) for both regions.

To explore the evolutionary patterns among closely related species, we focused on 12 *Gtf* genes in clade I and divided these into four groups, with each group having orthologous *Gtf* genes from three closely related species. In each group, the protein sequence identities and the dN/dS ratios were calculated for GH70 domain and A repeat units. The protein sequence identity levels among multiple A repeat units in each enzyme were also examined.

## Results

### The Distribution of *Gtf* Genes Among *Streptococcus* Species in Different Groups

To clearly define the *Gtf* gene numbers among different *Streptococcus* species, a total of 872 genomes covering 37 *streptococcus* species were surveyed (Figure 1 and Supplementary Table S1). In the Mitis group, *Gtf* genes were not detected in six of the seven surveyed species, including 21 *S. mitis* genomes, 47 *S. pneumoniae* genomes, 10 *S. infantis* genomes, one *S. peroris* genome, two *S. australis* genomes, and 18 *S. parasanguinis* genomes (Figure 1). Since multiple completely assembled genomes were examined for species *S. mitis, S. pneumoniae*, and *S. parasanguinis* (Supplementary Table S1), it is safe to conclude that the *Gtf* gene is absent from these species. In *S. oralis*, one type of *Gtf* gene, namely *GtfR*, was detected in 16 out of 30 examined genomes (Figure 1 and Supplementary Table S1). In the Sanguinis group, *Gtf* genes were detected from 21 out of 26 *S. gordonii* genomes, three out of 15 *S. cristatus* genomes and 28 out of 30 *S. sanguinis* genomes (Figure 1). The high detection rates of *Gtf* genes in species *S. gordonii* (81%) and *S. sanguinis* (93%) justify the presence of *Gtf* gene in both species, but the detection rate (20%) in *S. cristatus* is rather low. Interestingly, in a completely sequenced genome of *S. cristatus* strain AS 1.3089 (NC_021175), no *Gtf* gene was found (Supplementary Table S1). In the Anginosus group, a total of 27, 12, and 15 genomes were examined for *S. anginosus, S. constellatus*, and *S. intermedius*, respectively, but the *Gtf* gene was not detected (Figure 1 and Supplementary Table S1). Similarly, the *Gtf* gene was absent in 35 genomes of the independent species, *S. suis*.

Among a total of 235 genomes examined for eight species of the Pyogenic group, only one short *Gtf* gene was detected in the *S. urinalis* strain 2285-97 genome (Figure 1 and Supplementary Table S1). Since many completely sequenced genomes were examined for *S. dysgalactiae, S. pyogenes, S. equi, S. uberis, S. iniae*, and *S. agalactiae*, the absence of the *Gtf* gene can be justified for these species. However, this was not the case for the Bovis group. Among the three surveyed species, *Gtf* genes were detected in 17 out of 20 *S. gallolyticus* genomes, 26 out of 31 *S. equinus* genomes, and 2 out of 3 *S. infantarius* genomes (Figure 1 and Supplementary Table S1). Two different types of the *Gtf* genes were seen in *S. gallolyticus*.

In the Mutans groups, six species of two subgroups were surveyed. Interestingly, three species in the first subgroup, *S. ratti, S. devrieseii*, and *S. orisasini*, all possessed a maximum of two different types of *Gtf* genes, while three species in the other subgroup, *S. mutans, S. troglodytae*, and *S. macacae*, all contained a maximum of three types of *Gtf* genes (Figure 1 and Supplementary Table S1). In the Downei group, 49 genomes representing three species, *S. criceti, S. downei*, and *S. sobrinus*, were examined and a maximum of five types of *Gtf* genes were detected from each species. Finally, in the Salivarius group, no *Gtf* gene was found from 33 *S. thermophilus* genomes and six *S. vestibularis* genomes. However, among 13 *S. salivarius* genomes examined, up to six types of *Gtf* genes were identified (Figure 1 and Supplementary Table S1).

Overall, our survey identified 45 types of *Gtf* genes from 18 out of 37 investigated *Streptococcus* species (Figure 1). More than half of the surveyed species contained no *Gtf* gene, and these species were distributed in the Mitis, Anginosus, Pyogenic, and Salivarius groups. Seven species, including *S. oralis* of the Mitis groups, three species of the Sanguinis group, *S. urinalis* of the Pyogenic group, and two species of the Bovis group, possessed only one *Gtf* gene. The other 11 species, including *S. gallolyticus* of the Bovis group, *S. salivarius* of the Salivarius group, and all nine surveyed species of the Mutans and Downei groups, possessed two to six types of *Gtf* genes in their genomes (Figure 1 and Supplementary Table S1).

### Establishing an Elaborate and Robust *Gtf* Gene Phylogeny in *Streptococcus*

**FIGURE 2:**
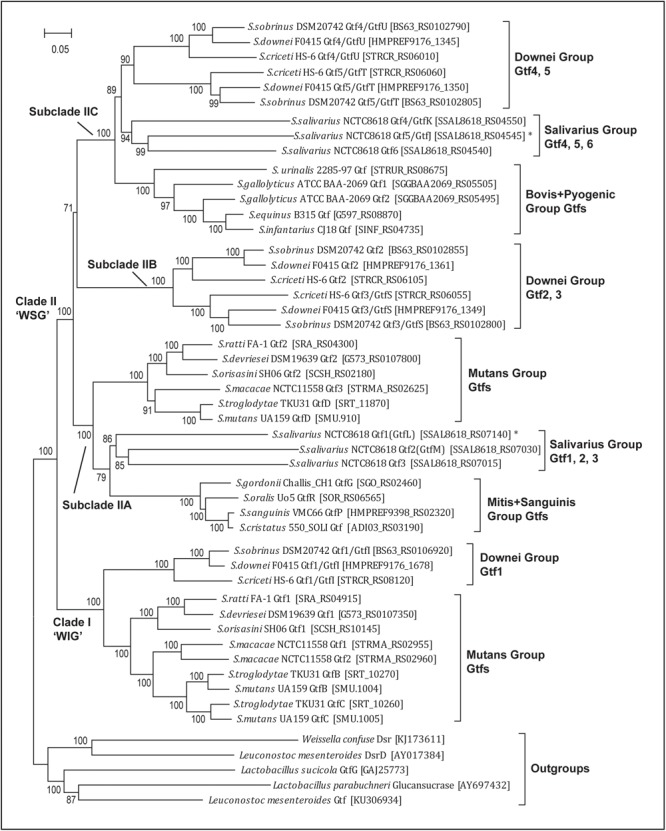
Phylogenetic analysis of 45 streptococcal glucosyltransferase (*Gtf*) genes reveals one WIG clade (I) and three WSG subclades (IIA, IIB, and IIC). WIG or WSG refers to clades in which *Gtf* genes are mainly responsible for synthesizing water-insoluble glucans or water-soluble glucans. The tree was built with the Maximum-Likelihood method using the aligned homologous sequences (∼3.9 kb) of *Gtf* genes. Five homologous sequences from *Weissela, Lactobacillus* and *Leuconostoc* were chosen as outgroups. The bootstrap support values from 100 replicates are shown for each internal branch and the bar represents 0.05 substitutions per nucleotide position. Note the WIG clade is specific for nine species of two caries-promoting groups, and two *Streptococcus salivarius* genes (*Gtf1*/*GtfL* and *Gtf5*/*GtfJ*, marked with asterisks) known to synthesize insoluble glucans are nested within WSG subclades IIA and IIC, respectively.

We then established an updated phylogeny for the *Streptococcus Gtf* genes. By selecting one representative strain for each of the 18 species, a total of 45 *Gtf* genes were compiled and aligned with the five outgroup sequences. After removing two variable regions that are not suitable for phylogenetic inferences, the remaining 3.9 kb alignment (for details, see section “Materials and Methods”) was used to build a highly robust *Streptococcus Gtf* gene phylogeny, which received strong bootstrap support for all internal branches (Figure 2).

The updated phylogeny supported two previous findings. First, since all 45 sequences formed a monophyletic group, *Streptococcus Gtfs* indeed share a common ancestry. Second, the phylogeny includes two major clades, one WIG clade and the other WSG clade (Figure 2). Clade I includes *Gtf* genes from nine caries-promoting species from the Mutans and Downei groups, and their encoded enzymes are known to synthesize WIG that are critical for modifying biofilm structures. Clade II contains *Gtf* genes from all 18 species, and these genes are mainly responsible for synthesizing WSG.

The new phylogeny further clarified a more elaborate relationship within both clades. In the WIG clade, the *Gtf1*/*GtfI* genes from three Downei group species formed an independent branch, while the *Gtf* genes from six Mutans group species also grouped together, forming a branch sister to the Downei group *Gtf* genes. Within the Mutans *Gtf* branch, the *Gtf1* genes from *S. ratti, S. devriesei*, and *S. orisasini* clustered together, forming a sub-branch, while the *Gtf* genes from the other three species, *S. macacae, S. troglodytae*, and *S. mutans*, formed the other sub-branch. In the WSG clade, the topology is much more complicated, having diverged into at least three subclades (IIA, IIB, and IIC, Figure 2). Different *Streptococcus* groups have inherited these subclades differentially. The subclade IIA includes 11 species from the Mutans, Salivarius, Mitis, and Sanguinis groups, while the topology revealed a closer relationship between three *S. salivarius* genes (*Gtf1-3*) and four *Gtf* genes from the Mitis and Sanguinis group species. The subclade IIB includes only the three Downei group species, while its sister lineage, subclade IIC, includes eight species from not only the Downei group, but also from the Salivarius, Bovis, and Pyogenic groups. Consisting with the species phylogeny, the only identified *Gtf* gene from the Pyogenic group species, *S. urinalis* 2285-97 *Gtf*, was found diverged earlier than the four *Gtf* genes from the three Bovis group species. Moreover, the sister relationship between the Downei group and Salivarius group is also supported by the gene phylogeny of subclade IIC (Figure 2).

The new *Gtf* gene phylogeny helped to define multiple duplication events (Figure 2). In clade I, gene duplications occurred in three Mutans group species, *S. macacae, S. troglodytae*, and *S. mutans*, resulting in two copies of clade I *Gtf* genes in these species. In clade II, duplication events occurred more frequently. Successive duplications occurred in *S. salivarius*, resulting in three copies (*Gtf1-3*) in subclade IIA and another three copies (*Gtf4-6*) in subclade IIC. The three Downei group species seem to share a common duplication history, including an ancient duplication event giving rise to subclades IIB and IIC and two subsequent duplications, one producing the *Gtf2* and *Gtf3*/*GtfS* pair in subclade IIB and the other producing the *Gtf4*/*GtfU* and *Gtf5*/*GtfT* pair in subclade IIC (Figure 2). Examining the genomic locations of these four *Gtf* genes in the three Downei group species also revealed a conserved pattern. In addition, a gene duplication event also occurred on the species *S. gallolyticus*, resulting in two genes (*Gtf1* and *Gtf2*) in subclade IIC.

### Reconstructing *Gtf* Gene Phylogenies Using Data From Multiple Strains

To examine whether the selected representative strain is appropriate for each *Streptococcus* species and to verify the *Gtf* gene relationships within each clade or subclade, we further reconstructed *Gtf* gene phylogenies using sequence data from multiple strains (Supplementary Table S1 and Supplementary Figure S1). The clade I phylogeny (Supplementary Figure S1A) includes 43 *Gtf* genes from 30 strains of nine species, and the topology is in total agreement with that reported in Figure 2, with *Gtf1* genes from 11 *S. sobrinus* strains and *GtfB* and *GtfC* genes from 11 *S. mutans* strains all grouped together on their own. Up to 122 *Gtf* genes from 98 stains of 11 species were used to reconstruct the subclade IIA phylogeny (Supplementary Figure S1B). Interestingly, when compared to the topology reported in Figure 2, three *S. cristatus Gtf* genes are not grouped together. The *Gtf* gene from the representative strain *S. cristatus* 550_SOLI is nested within the *S. sanguinis GtfP* group, while the other two detected *Gtf* genes from *S. cristatus* strains 771_SOLI and 787_SOLI were found within the *S. gordonii GtfG* group. Such results suggest that the three strains were likely misidentified as *S. cristatus* for genomic sequencing. If so, there would be no real *Gtf* gene identified from *S. cristatus*, which may explain why no *Gtf* gene could be found from a completely sequenced genome of *S. cristatus* strain AS 1.3089, as mentioned earlier.

Supplementary Figure S1C presents the reconstructed subclade IIB phylogeny using a total of 26 *Gtf* genes from 13 strains of three Downei group species. The topology is the same as that in Figure 2. The subclade IIC phylogeny included a total of 126 *Gtf* genes from 72 strains of eight species (Supplementary Figure S1D) and the overall within-clade relationships were confirmed. The only abnormality was that two *S. infantarius Gtf* genes, although grouped together, were found at an intermediate position between two subgroups of *S. equinus Gtf* genes. The branches leading to the two subgroups of *S. equinus* were longer than cases of other *Streptococcus* species, indicating a tendency of divergence in this species.

### The Overall Evolutionary Patterns of *Streptococcus Gtfs*

After clarifying the evolutionary relationships of *Streptococcus Gtf* genes, we explored how selection pressures had shaped these genes. Duplicated *Gtf* genes were examined first. According to the phylogeny (Figure 2), a total of 16 pairs of *Gtf* genes involving eight species were compared (Table 1). The variant branch lengths leading to these gene pairs indicated that these duplication events had occurred at different time points during the *Gtf* gene evolutionary history. The full gene divergence levels, D*_*gene*_*, ranged from 0.243 for *S. mutans GtfB-GtfC* to 0.650 for *S. salivarius Gtf1-Gtf2* (Table 1). For the three Downei group species sharing a common duplication history, the calculated D*_*gene*_* values were also similar (0.427, 0.440, and 0.480 for *Gtf2-Gtf3* and 0.545, 0.534, and 0.529 for *Gtf4-Gtf5*). The calculated D*_*gene*_* values for the *GtfB*-*GtfC* pair in *S. troglodytae* and *S. mutans* were also close, being 0.255 and 0.243, respectively (Table 1). When examining different domains, the conserved GH70 domain exhibited smaller genetic distances between duplicated genes, with D*_*GH70*_* values ranging from 0.059 to 0.532, while the C-terminal repeat regions showed high variations, with D*_*repeat regions*_* values ranging from 0.353 to 0.971. Evaluation of the dN/dS ratios revealed values for the GH70 domain of 0.22 to 0.67, suggesting this domain was subjected to purifying selection. However, the values for the C-terminal repeat regions ranged from 0.60 to 1.03, indicating this region was subjected to more relaxed constraints than the GH70 domain.

**Table 1:** Divergence patterns of duplicated *Gtf* genes in multiple *Streptococcus* species.

Clade/ Subclade	Species	*Duplicated Gtf* genes	D*_*gene*_*	GH70 domain		C-terminal repeat region	
				D*_*GH70*_*	Dn/Ds	D*_*repeat region*_*	Dn/Ds
Clade I	*S. mutans*	*GtfB-GtfC*	0.243	0.059	0.29	0.530	0.60
	*S. troglodytae*	*GtfB-GtfC*	0.255	0.084	0.23	0.505	0.63
	*S. macacae*	*Gtf1-Gtf2*	0.274	0.104	0.33	0.437	0.70
Subclade IIA	*S. salivarius*	*Gtf1-Gtf2*	0.650	0.487	0.47	0.838	0.71
	*S. salivarius*	*Gtf1-Gtf3*	0.630	0.458	0.52	0.854	0.96
	*S. salivarius*	*Gtf2-Gtf3*	0.626	0.454	0.55	0.786	0.75
Subclade IIB	*S. criceti*	*Gtf2-Gtf3*	0.427	0.235	0.23	0.971	0.89
	*S. downei*	*Gtf2-Gtf3*	0.440	0.277	0.22	0.857	0.85
	*S. sobrinus*	*Gtf2-Gtf3*	0.480	0.297	0.25	0.891	0.97
Subclade IIC	*S. gallolyticus*	*Gtf1-Gtf2*	0.325	0.234	0.31	0.353	0.43
	*S. salivarius*	*Gtf4-Gtf5*	0.600	0.532	0.67	0.791	1.03
	*S. salivarius*	*Gtf4-Gtf6*	0.566	0.464	0.57	0.794	1.03
	*S. salivarius*	*Gtf5-Gtf6*	0.519	0.417	0.52	0.816	1.01
	*S. criceti*	*Gtf4-Gtf5*	0.545	0.437	0.47	0.753	0.83
	*S. downei*	*Gtf4-Gtf5*	0.534	0.423	0.51	0.735	0.87
	*S. sobrinus*	*Gtf4-Gtf5*	0.529	0.420	0.49	0.744	0.83

*D_*gene*_, genetic distances between paired genes; D_*GH70*_, genetic distances of paired genes for the conserved glycoside hydrolase family 70 domain; D_*repeat region*_, genetic distances of paired genes for the C-terminal variable repeat regions. Dn, non-synonymous substitution rate; Ds, synonymous substitution rate. The Tamura-Nei model was used to calculate the genetic distances and the modified Nei-Gojobori method (Proportion) was used to calculate the Dn and Ds values.*

### The C-Terminal Repeat Region of Gtf Enzymes Exhibited Dynamic Evolutionary Patterns

**FIGURE 3:**
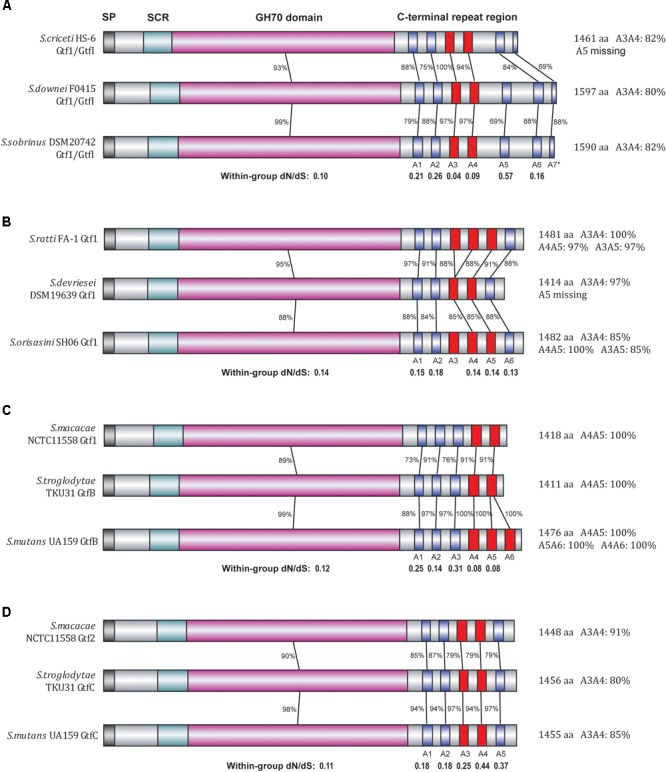
Divergence patterns of 12 Gtf enzymes of clade I. The 12 Gtf enzymes were divided into four groups **(A–D)** according to their phylogenetic relationships, with their domain structures drawn in different colors. SP, signal peptide region (black); SCR, short conserved region (light blue); GH70 domain (pink), A repeat units (blue). Highly similar A repeats (80% or higher amino acid sequence identities) were shown in red. In each group, the protein sequence identities and the dN/dS ratios were calculated for the GH70 domain as well as for each individual A repeat unit.

To further explore the evolutionary patterns at the C-terminal repeat region, we carefully determined the numbers and positions of A repeat units present in each of the 45 representative *Streptococcus* Gtf enzymes as well as the five outgroup enzymes (Supplementary Table S2). Most of these enzymes possess 5 to 7 A repeat units, although in some cases the last A repeat unit was truncated. For example, the *S. downei* Gtf1/GtfI enzyme possesses six intact A repeat units (A1–A6) as well as a 16-aa partial A7 unit at the C-terminal end. Interestingly, for duplicated Gtfs, the numbers of repeat units are often different. For example, the successively duplicated *S. salivarius* Gtf 1–3 have 4, 6, and 7 repeat units, respectively, while the *S. gallolyticus* Gtf1 (A1–A7) has one more repeat unit than the duplicated Gtf2 (A1–A6). To determine how the repeating unit numbers have changed among different Gtfs, we further compared the sequence homology levels among different A repeat units of the 50 enzymes. Using 80% identity as a cutoff, highly similar A repeat units in terms of their amino acid sequences were found in 18 *Streptococcus* Gtf enzymes as well as two *Lactobacillus* enzymes (Supplementary Table S2). Interestingly, all 12 of the clade I Gtf enzymes were included in this category, with two or three tandem A repeats sharing 80–100% identities (Figure 3). For example, among the six A repeat units in *S. mutans* GtfB enzyme, A4, A5, and A6 showed 100% amino acid sequence identities.

We then focused on these clade I Gtfs to examine the evolutionary patterns of each individual A repeat. To conduct close comparisons, these Gtfs were divided into four groups (Figures 3A–D), with each group containing Gtfs from three closely related species. In group A, the Gtf1/GtfI enzymes from species *S. criceti, S. downei*, and *S. sobrinus* were studied (Figure 3A). The GH70 domain exhibited 93% amino acid sequence identities between *S. criceti* and *S. downei* and 99% identities between *S. downei* and *S. sobrinus*. Compared to the GH70 domain, the C-terminal repeat units A1, A2, and A5–A7 all exhibited lower identity levels and higher dN/dS ratios, and A5 was even lost from the *S. criceti* enzyme, suggesting these five repeat units have experienced fewer functional constraints than the GH70 domain (as revealed in the last section between duplicated gene pairs). However, for the A3 and A4 repeat units, high amino acid sequence identity levels have been surprisingly maintained among the three species. Between *S. criceti* and *S. downei*, the A3 unit showed 100% amino acid sequence identity, although the nucleotide identity was only 84%. The calculated dN/dS ratios for A3 (0.04) and A4 (0.09) are smaller than those of the GH70 domain (0.10), suggesting that these two repeat units have been suffering stronger functional constraints during the divergence of these three species.

In the other three groups, the homology levels and dN/dS ratios were all calculated for GH70 domain and individual A repeat units (Figures 3B–D). For the A2 repeat of group B, A1 and A3 repeats of group C and all five A repeats (A1–A5) of group D, lower homology levels and higher dN/dS ratios than their GH70 domains were observed, suggesting that it is easier for these repeat units to change their amino acids among closely related species. However, for repeats A4 and A5 in group C, higher identity levels were maintained and smaller dN/dS ratios (0.08) than GH70 domain (0.12) were observed, reflecting an evolutionary pattern of difficult to change their amino acid sequences among species.

## Discussion

Dental caries is now viewed as a biofilm-mediated sugar disease that occurs when acidogenic/aciduric microorganisms are selectively favored over other microorganisms, disrupting the original homeostasis of microbiota under healthy condition (Kilian et al., 2016; Sanz et al., 2017; Manji et al., 2018; Philip et al., 2018; Twetman, 2018). This disease affects both human and dentate mammals (Kohler, 2007). A number of *Streptococcus* species that were historically viewed as caries ‘pathogens’ of human or animals are actually present with low proportions in healthy biofilms (Bowen and Koo, 2011; Krzysciak et al., 2014; Kilian et al., 2016; Philip et al., 2018). Under disease condition, however, these *Streptococcus* species would become caries-promoting by synthesizing WIG from sucrose, which would modify the biofilm structure and allow fast fermentation of carbohydrates and acid production (Mukasa et al., 1985; Yamashita et al., 1993; Monchois et al., 1999; Bowen and Koo, 2011; Koo et al., 2013; Krzysciak et al., 2014; Guo et al., 2015; Kilian et al., 2016; Philip et al., 2018). Therefore, Gtf enzymes responsible for WIG synthesis are important for caries development. In this study, we investigated how *Gtf* genes have evolved in *Streptococcus*, with an emphasis on the evolutionary patterns of WIG-synthesizing *Gtf* genes in clade I.

By surveying 872 genomes covering 37 *Streptococcus* species from all of the major phylogenetic groups, we were able to detect 45 different types of *Gtf* genes from 18 species (Figure 1 and Supplementary Table S1). However, the distribution pattern of these *Gtf* genes between two major *Streptococcus* clades is strongly biased, with only four *Gtf* genes seen from the ASM clade (comprising of the Anginosus, Sanguinis, and Mitis groups) and 41 *Gtf* genes from the SDMBP clade (including the Salivarius, Downei, Mutans, Bovis, and Pyogenic groups, Figure 1). Such unbalanced distribution of *Gtf* genes in two major *Streptococcus* clades prompted us to consider when the ancestral *Gtf* gene originated in *Streptococcus*. According to two previous studies (Hoshino et al., 2012; Argimon et al., 2013), all *Streptococcus Gtf* genes have a common ancestry, suggesting that the ancestral *Gtf* gene originated in the common ancestor of all current *Streptococcus* species. However, considering the distribution patterns of *Gtf* genes revealed by this study (Figure 1), an alternative hypothesis could also be raised; namely, that the ancestral *Gtf* gene originated in the common ancestor of the SDMBP clade, while the *Gtf* genes found in a few species in the ASM clade were acquired secondarily.

To clarify which hypothesis is more likely to be true, a reliable and elaborate *Streptococcus Gtf* gene phylogeny is required. In this study, by using all 45 *Gtf* genes identified from the 18 *Streptococcus* species, we established an updated *Gtf* gene phylogeny that received strong bootstrap support for all of the internal branches (Figure 2). It should be emphasized that the alignment (∼3.9 kb) used to reconstruct this robust ML tree includes four rather conserved regions, the N-terminal signal peptide region (SP), a short conserved region (SCR) upstream of the central catalytic GH70 domain, the entire GH70 domain, and the first two glucan-binding domain repeat units at the C-terminal repeat region (Supplementary Table S2). Previous studies only used the conserved GH70 domain region to reconstruct *Gtf* gene phylogenies (Hoshino et al., 2012; Argimon et al., 2013). However, the range of the GH70 domain is often not clearly defined. In this study, by using the NCBI conserved domain search website (see section “Materials and Methods”), we defined the range of the GH70 domain (pfam02324) as sites 287 to 1067 (corresponding to sites 269 to 1047 in *S. mutans* GtfB, see Supplementary Table S2). Immediately upstream of the GH70 domain, a short conserved region (SCR, ∼100 aa) was also identified. In fact, this short region was previously taken as the beginning part of the GH70 domain and used for phylogenetic analysis (Giffard et al., 1993; Argimon et al., 2013). However, the C-terminal repeat region was never used for phylogenetic inferences before this study. By carefully determining the ranges of each glucan-binding repeat unit among 50 Gtfs (Supplementary Table S2) and examining their similarity levels, the first two repeat units were found to share high sequence homology among all Gtfs. To determine if these two repeat units could also provide authentic signals for phylogenetic inference, we also built a phylogeny using a 3.1 kb alignment with only the SP, SCR, and GH70 domain regions included. The obtained phylogeny (available upon request) was the same as that in Figure 2 except in subclade IIB, where two *S. criceti Gtfs* (*Gtf2* and *Gtf3*) were grouped together, forming a separate cluster. Moreover, if the SP and SCR regions were further precluded and only the GH70 domain region was used, the obtained phylogeny (available upon request) showed two more differences; namely, in clade I, the *S. troglodytae GtfB* and *GtfC* and *S. mutans GtfB* and *GtfC* formed separate clusters, while in subclade IIA, three *S. salivarius Gtfs* moved to the basal position with low bootstrap support. Therefore, both the SCR region and the first two repeat units likely provide extra phylogenetic signals. One likely scenario is that for duplicated genes (*Gtf2–Gtf3* or *GtfB–GtfC*) in species such as *S. criceti, S. troglodytae*, and *S. mutans*, recombination events probably occurred in the conserved GH70 domain, tending to bring them together when only the GH70 domain was used for phylogenetic reconstruction. However, the real topology is more likely to be obtained when more homologous regions, such as SCR and the first two repeat units, are included. Giffard et al. (1993) presented evidence supporting that partial recombination had occurred between *S. mutans GtfB* and *GtfC* genes in the GH70 domain, with a gene distance much higher in block 1 (matching the SCR region in this study) than in other regions of the GH70 domain. In fact, recombination events have likely occurred multiple times during the *Streptococcus Gtf* gene evolution, and one should be cautious in explaining the reconstructed phylogenetic relationships if the GH70 domain is used. For example, in the updated phylogeny of this study, the two *S. macacae Gtf* genes in clade I grouped together with 100% bootstrap support, suggesting these two genes were duplicated in *S. macacae*. However, the grouping of the two *S. macacae* genes with the *GtfB* and *GtfC* genes of the species *S. troglodytae* and *S. mutans* was supported in a previous study when full length amino acid sequences were used to build the phylogeny (Argimon et al., 2013). Indeed, when the SCR and C-terminal repeat regions were examined between *S. macacae* Gtf1 and Gtf2, the SCR region showed only 57% and the A1 to A3 repeat units exhibited 66–73% identities, while their GH70 domains were 91% identical (indicating recent recombination in this region). Therefore, it is more likely that *S. macacae Gtf1* share a common ancestor with *S. troglodytae* and *S. mutans GtfB* genes, so as the *S. macacae Gtf2* with *S. troglodytae* and *S. mutans GtfC* genes.

The updated phylogeny was then used to determine when the ancestral *Gtf* gene originated in *Streptococcus*. The WIG clade only contains *Gtf* genes from nine species of the Downei and Mutans groups, and the gene phylogeny is consistent with the species phylogeny, suggesting that the ancestral clade I *Gtf* gene must have appeared in a common ancestor of the Downei and Mutans groups, that is a common ancestor of the SDMBP clade if the *Streptococcus* phylogeny in Figure 1 is accepted. These findings further indicate that the ancestral clade I *Gtf* gene have likely been lost from three other groups of this clade, including the Salivarius, Bovis, and Pyogenic groups. The WSG clade includes 18 species from seven phylogenetic groups, and we proposed that this clade had further diverged into three subclades (IIA, IIB, and IIC). Subclade IIC contains *Gtf* genes from species of four groups (Downei, Salivarius, Bovis, and Pyogenic), and the phylogeny mirrors the species phylogeny, with just the Mutans branch missing. These findings indicate that the ancestral subclade IIC gene had appeared in a common ancestor of the SDMBP clade. Since subclade IIB is a sister to subclade IIC, this branch must also have originated from a common ancestor of the SDMBP clade. Subclade IIA includes *Gtf* genes from species of not only the Mutans and Salivarius groups in the SDMBP clade, but also from the Mitis and Sanguinis groups of the ASM clade. However, the *Gtf* gene topology here is not consistent with the species phylogeny. Instead of being placed outside of the Mutans and Salivarius groups, the four *Gtf* genes from the Mitis and Sanguinis group species showed a close relationship with *S. salivarius Gtf1-3* genes. Furthermore, a previous study by Hoshino et al. (2012) revealed the presence of transposase-like sequences around the *S. oralis GtfR* gene. If one assumes that the ancestral *Gtf* gene originated in the common ancestor of *Streptococcus* SDMBP clade, then the *Gtf* genes found in *S. oralis* and three Sanguinis species would more likely have been acquired secondarily from a Salivarius group species. However, if one assumes that the ancestral *Gtf* gene originated in an ancestor common to all current *Streptococcus* species, the new *Gtf* gene phylogeny updated by this study must be explained by multiple independent losses of *Gtf* genes in the ASM clade.

In this study, we also put great effort into analyzing the evolutionary patterns of *Gtf* genes, especially for the variable C-terminal repeat region (Supplementary Table S2 and Figure 3). This region is known to include multiple numbers of glucan-binding domain (GBD) repeats, which have the potential to bind glucans (Monchois et al., 1999; Leemhuis et al., 2013). Early studies have identified several repeat unit sequences from the repeat region of various Gtf enzymes, including A, B, C, D, and YG repeats (Ferretti et al., 1987; Banas et al., 1990; Gilmore et al., 1990; Giffard et al., 1991, 1993: Giffard and Jacques, 1994; Shah et al., 2004). Among these, the A repeat unit (∼33 aa) represents the most conserved sequence and is also widely present among various Gtfs. In the present study, 3–7 A repeat units were identified from 45 representative Gtf enzymes. Evaluation of multiple pairs of duplicated *Gtf* genes revealed that the C-terminal repeat region, as a whole, has been subjected to rather relaxed constraints compared to the GH70 domain (Table 1). However, careful analysis of individual A repeat units among closely related species revealed two contradictory evolutionary patterns (Figure 3). Many A repeats have been suffering relaxed evolutionary pressure and have experienced more amino acid changes. However, some A repeat units, such as A3 and A4 in Gtf1/GtfI of Downei group species, and A4 and A5 in GtfB of *S. macacae, S. mutans*, and *S. troglodytae*, have been subjected to strong functional constraints preventing amino acid changes (Figure 3). More interestingly, these functionally important A repeat units have often been duplicated in tandem in their *Gtf* genes. By deleting various lengths of 3′-ends from a *S. sobrinus Gtf1/GtfI* gene clone, Abo et al. (1991) revealed that when the A5 to A7 repeat units are completely removed, the expressed enzyme will still have normal glucan-binding function and be able to synthesize insoluble glucans. However, when both A3 and A4 are further removed, the enzyme only shows weak glucan-binding ability and is unable to synthesize insoluble glucans. Therefore, the A3 and A4 repeat units in Downei group Gtf1/GtfI enzymes are probably involved in synthesis of insoluble glucans, which are critical for the caries-promoting ability of these species. Similarly, the A4 and A5 repeats in *S. macacae* Gtf1 and *S. troglodytae* GtfB, and A4–A6 repeats in *S. mutans* GtfB need to be examined in future to reveal their functional relevance to insoluble glucan synthesis.

In summary, a comprehensive survey of *Gtf* genes among *Streptococcus* species was conducted. By building a robust *Gtf* gene phylogeny, a greater understanding of the origination and evolutionary history of *Streptococcus Gtf* genes among various phylogenetic groups was obtained. Also, by performing a thorough analysis of the C-terminal repeat regions, we revealed two different evolutionary patterns for those individual A repeat units. The A repeat units subjected to strong functional constraints should be investigated further because they are probably involved in synthesizing caries-promoting, water-insoluble glucans.

## Author Contributions

R-RX, BW, and W-MW conceived and designed the project. R-RX, W-DY, and BW collected the data and performed relevant analyses. R-RX, K-XN, and BW organized the tables and figures. R-RX, BW, and W-MW wrote the manuscript. BW and W-MW supervised the project.

## Conflict of Interest Statement

The authors declare that the research was conducted in the absence of any commercial or financial relationships that could be construed as a potential conflict of interest.

## Footnotes

1 https://www.ncbi.nlm.nih.gov/genome/microbes/

2 https://www.ncbi.nlm.nih.gov/Structure/cdd/wrpsb.cgi

## Supplementary Material

TABLE S1 The detected Glucosyltransferase (*Gtf*) genes among 872 *Streptococcus* genomes surveyed by this study.

TABLE S2 The identified domains in 50 Gtf proteins. SP, the N-terminal signal peptide region (Honda et al., 1990); SCR, a short conserved region before the GH70 domain; GH70, the Glycoside Hydrolase family 70 domain (pfam02324); A1–A7, the identified A repeat units (∼33aa, Gilmore et al., 1990; Shah et al., 2004) present in each Glucan-binding domain (GBD, TIGR04035, 65aa) at the C-terminal variable region. Partial domains were shown in italic and marked with asterisks.

FIGURE S1 The reconstructed Maximum-Likelihood trees of *Streptococcus* glucosyltransferase (*Gtf*) genes for each clade or subclade using sequence data from multiple strains. **(A)** The reconstructed clade I phylogeny using 43 *Gtf* genes from 30 strains of 9 species; **(B)** the subclade IIA phylogeny including 122 *Gtf* genes from 98 strains of 11 species (note the three *Streptococcus cristatus* sequences marked with asterisks are grouped with either the *S. gordonii* or *S. sanguinis* sequences); **(C)** the subclade IIB phylogeny comprising of 26 *Gtf* genes from 13 strains of 3 species; **(D)** the subclade IIC phylogeny covering 126 *Gtf* genes from 72 strains of 8 species (note the two *S. infantarius* sequences marked with asterisks are nested within the *S. equinus* sequences). In each phylogeny, two homologous sequences from *Lactobacillus* and *Leuconostoc* are used as outgroups. The bootstrap support values from 100 replicates are shown for each internal branch and the bar represents 0.1 substitutions per nucleotide position.
