# Supplementary figures and images for: An Update on the Evolution of Glucosyltransferase (Gtf) Genes in Streptococcus
Source: Front Microbiol. 2018 Dec 4;9:2979. doi: 10.3389/fmicb.2018.02979 (PMC6290343; doi:10.3389/fmicb.2018.02979)

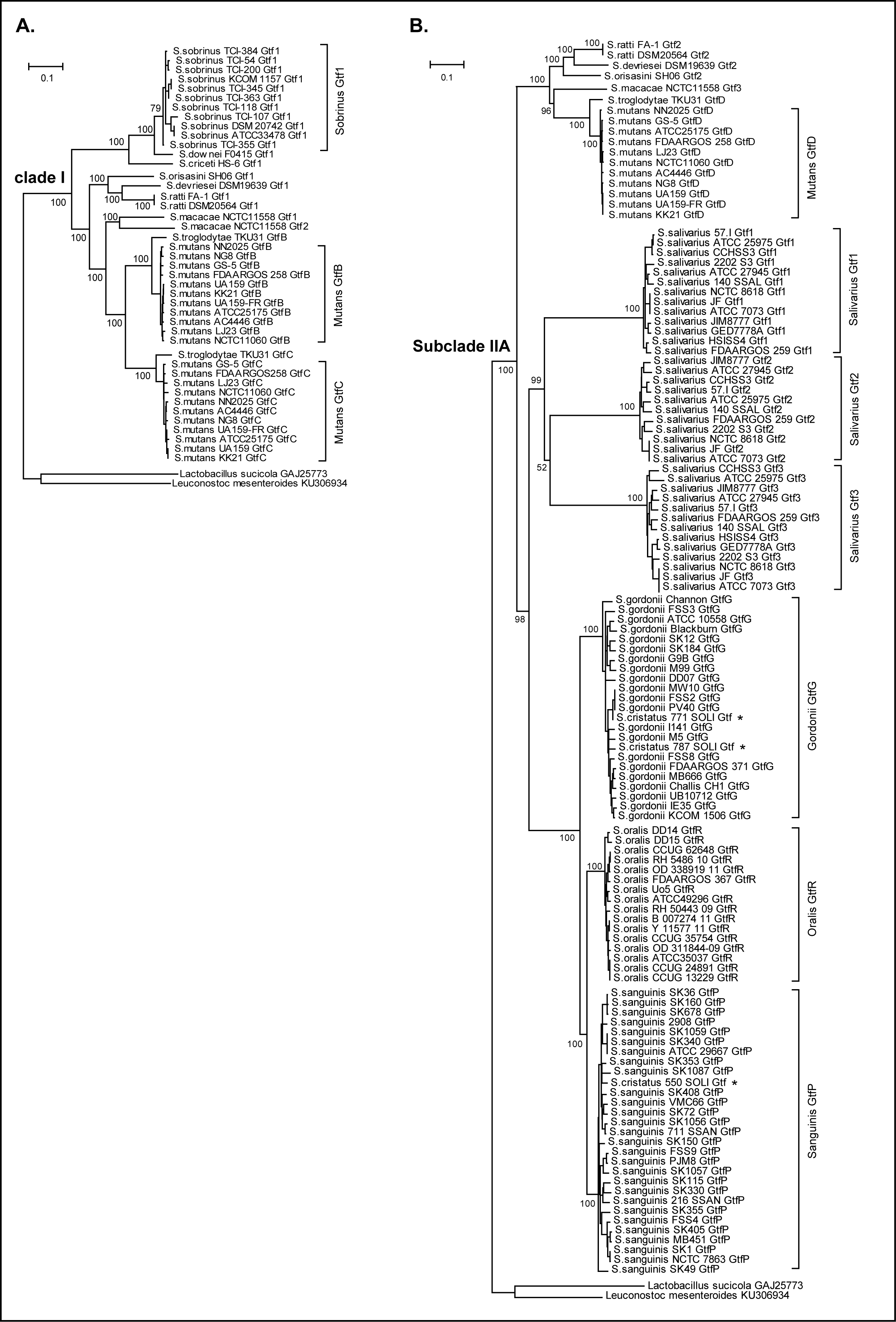

Supplement: FIGURE S1 — The reconstructed Maximum-Likelihood trees of Streptococcus glucosyltransferase (Gtf) genes for each clade or subclade using sequence data from multiple strains. (A) The reconstructed clade I phylogeny using 43 Gtf genes from 30 strains of 9 species; (B) the subclade IIA phylogeny including 122 Gtf genes from 98 strains of 11 species (note the three Streptococcus cristatus sequences marked with asterisks are grouped with either the S. gordonii or S. sanguinis sequences); (C) the subclade IIB phylogeny comprising of 26 Gtf genes from 13 strains of 3 species; (D) the subclade IIC phylogeny covering 126 Gtf genes from 72 strains of 8 species (note the two S. infantarius sequences marked with asterisks are nested within the S. equinus sequences). In each phylogeny, two homologous sequences from Lactobacillus and Leuconostoc are used as outgroups. The bootstrap support values from 100 replicates are shown for each internal branch and the bar represents 0.1 substitutions per nucleotide position. [file Image_1.TIF]

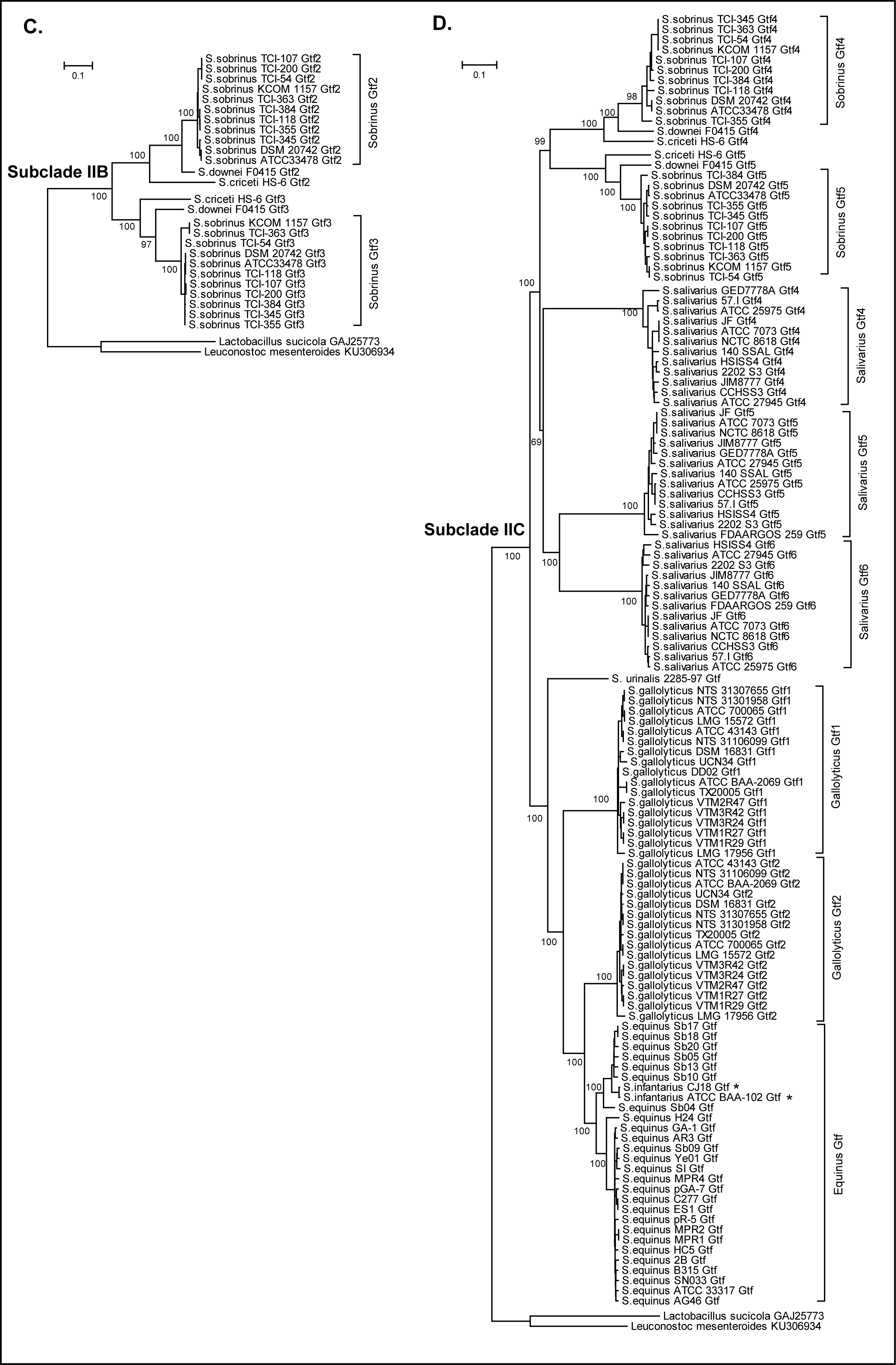

Supplement: Supplementary file 4 [file Image_2.TIF]
